# Vulnerabilities in *Yersinia pestis caf* Operon Are Unveiled by a *Salmonella* Vector

**DOI:** 10.1371/journal.pone.0036283

**Published:** 2012-04-30

**Authors:** Ling Cao, Timothy Lim, SangMu Jun, Theresa Thornburg, Recep Avci, Xinghong Yang

**Affiliations:** 1 Immunology and Infectious Diseases, Montana State University, Bozeman, Montana, United States of America; 2 Imaging and Chemical Analysis Laboratory, Department of Physics, Montana State University, Bozeman, Montana, United States of America; Indian Institute of Science, India

## Abstract

During infection, *Yersinia pestis* uses its F1 capsule to enhance survival and cause virulence to mammalian host. Since F1 is produced in large quantities and secreted into the host tissues, it also serves as a major immune target. To hold this detrimental effect under proper control, *Y. pestis* expresses the *caf* operon (encoding the F1 capsule) in a temperature-dependent manner. However, additional properties of the *caf* operon limit its expression. By overexpressing the *caf* operon in wild-type *Salmonella enterica* serovar Typhimurium under a potent promoter, virulence of *Salmonella* was greatly attenuated both *in vitro* and *in vivo*. In contrast, expression of the *caf* operon under the regulation of its native promoter exhibited negligible impairment of *Salmonellae* virulence. In-depth investigation revealed all individual genes in the *caf* operon attenuated *Salmonella* when overexpressed. The deleterious effects of *caf* operon and the *caf* individual genes were further confirmed when they were overexpressed in *Y. pestis* KIM6+. This study suggests that by using a weak inducible promoter, the detrimental effects of the *caf* operon are minimally manifested in *Y. pestis*. Thus, through tight regulation of the *caf* operon, *Y. pestis* precisely balances its capsular anti-phagocytic properties with the detrimental effects of *caf* during interaction with mammalian host.

## Introduction

Plague is an ancient disease, responsible for 200 million deaths worldwide and still exists in parts of the world today [1], [2]. Plague is caused by the gram-negative bacterium *Yersinia pestis* carried by rodents and spread by a flea vector [2]. Plague transmitted to humans by the bite of infected fleas leads to the bubonic form of the disease [3], which can result in 50% mortality if left untreated [4]. Some individuals bitten by infected fleas will not develop bubonic plague, but rather develop septicemic plague, a more lethal form [5]. Although neither bubonic nor septicemic plague is contagious, a small minority of patients develop secondary pneumonic plague. Pneumonic plague is highly contagious since the distance required for effective aerosol transmission is 2 meters [2]. Pneumonic plague can cause 100% mortality if not treated and 50% mortality when antimicrobial treatment begins within 20 hours of the onset of symptoms [5]. To date, endemic areas include China, central and southern Africa, vast areas of Asia and South America, and the southwest portion of the United States. In addition, plague has been classified as a re-emerging disease by the World Health Organization [1].

Two types of clinical isolates of *Y. pestis* have been disclosed: the F1 capsular positive and negative strains. Although the F1 capsule has been shown to be required for successful transmission from the flea to the mammalian host [6], contradictory findings regarding its requirement for mammalian infection have been reported [7], [8]. A more recent elaborate study comparing the *caf* operon deletion mutant with its parent wild-type (wt) strain *Y. pestis* CO92 showed that *caf* is required for maintaining virulence in mice although it depends on the mouse background [9], suggesting the F1 capsule is an important virulence factor. However, in order to utilize the F1 capsule to assist in infection, the pathogen must reduce exposure of F1 capsular protein to the mammalian immune system since it is also a protective antigen [10]. To minimize the exposure of F1 proteins, the pathogen has adopted a strategy of placing *caf* operon expression under tight control of a temperature-sensitive promoter [11], such that, at mammalian body temperature, the *caf* operon becomes activated to produce virulence effects, i.e., anti-phagocysis [12]. It can be inferred that the advantage gained from the anti-phagocytic properties of F1 outweighs the potential disadvantage of immunogenicity, since F1 capsular positive strains are more frequently isolated in nature than F1 capsular negative strains [13]. Additionally, bubonic plague symptoms usually appear after 2–8 days of exposure to the bacteria, while pneumonic plague has an incubation period of 1–6 days [14]. Such a short time from incubation to disease does not allow for protective immune responses to develop against the F1 capsule.

Since the F1 capsule provides an overall beneficial effect for *Y. pestis*, we questioned why it does not utilize a potent constitutive promoter rather than an inducible one. We therefore hypothesized that other vulnerabilities associated with *caf* operon that may not allow its expression beyond a definite threshold. To investigate the potential vulnerabilities, we overexpressed *caf* in wt *Salmonella enterica* serovar Typhimurium to determine whether overexpression of *caf* would display any impact on the bacterial host. Results show that overexpression of either the *caf* operon or any genes within this operon are able to dramatically debilitate *Salmonella* virulence.

## Results

### Construction of recombinant *S*. Typhimurium H71 strains that overexpress the F1 capsule

**Figure 1:**
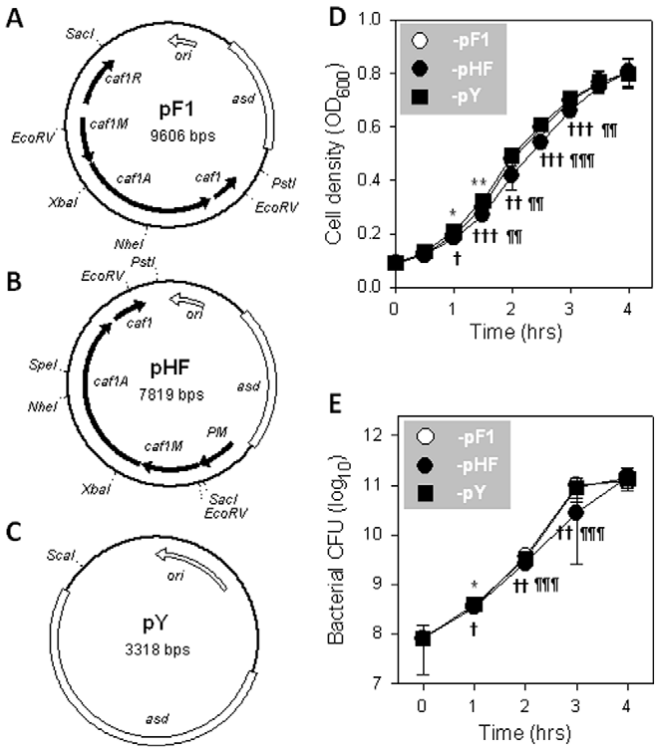
Schematic maps of plasmid pF1, pHF, and pY and growth rates of *Salmonella* strains harboring these plasmids. (**A**) In pF1, the *caf* operon is regulated by its native promoter P*caf1M*. (**B**) In pHF, the *caf* operon is regulated by a fusion promoter pM. (**C**) Plasmid pY is an empty vector. (**D, E**) Comparison of growth rates of P1-pF1, pHF, and pY. The bacterial growth rates were determined by measuring the OD_600_ every half hour (**D**) or determining bacterial CFU every hour (**E**), and the statistical differences were analyzed using the Tukey Kramer multiple comparisons test with * *P*<0.05, ** *P*<0.01, and *** *P*<0.001 for P1-pF1 vs -pY; ^†^ *P*<0.05, ^††^ *P*<0.01, and ^†††^ *P*<0.001 for -pHF vs -pY; and ^¶^ *P*<0.05, ^¶¶^ *P*<0.01, and ^¶¶¶^ *P*<0.001 for -pHF vs -pF1. Depicted are mean ± SEM of three independent experiments.

To allow stable maintenance and expression of the *caf* operon, the *asd*-mediated balanced-lethal system was utilized in this study as previously described [15]. Plasmid pF1, pHF and pY containing *asd* genes were utilized (Table 1 and Figure 1). In plasmid pF1, *caf* operon is regulated by its native promoter upstream of *caf1M* (P*caf1M*) and its own regulator *caf1R* [10]. Plasmid pHF was constructed by replacing the P*caf1M* in pF1 with a strong constitutive fusion promoter P*tetA*∼P*pagC*∼P*phoP* (PM) [16]. Plasmid pY is an empty vector that was used as a control. The *asd* mutation strain Δ*asd*::*kan* ^R^ *S.* Typhimurium H71 (P1) [16] was transformed with pF1, pHF, and pY, respectively, to obtain the recombinant strains P1-pF1, -pHF and -pY. Control strain P1-pY recovered full virulence to wt strain H71 in mice [17].

**Table 1:** Bacterial strains, plasmids, and their characteristics.

Bacterial strains	Phenotypes and characteristics	Sources or references
*E. coli* H681	*asd* ^−^. derived from parent strain X6212.	[10]
*S.* Typhimurium H71	Wild-type strain of *S.* Typhimurium.	[42]
*S.* Typhimurium P1	Δ*asd*::*kan* ^R^ H71.	[16]
*Y. pestis* KIM6+	*pgm* ^+^, *pst* ^+^, *lcr* ^−^, *fra* ^+^. derived from parent strain KIM-10.	[43]
Plasmids		
pJGX15C-*asd*	*asd* ^+^, *cfa/I* ^+^.	[44]
pHC	*asd* ^+^, *cfa/I* ^+^. The P*tetA* in pJGX15C-*asd* was replaced by fusion promoter PM.	[16]
pY	*asd* ^+^. *cfa/I* operon was removed from pJGX15C-*asd*.	This study
pF1	*asd* ^+^, operon *caf* ^+^. operon *caf* regulated by P*caf1M*.	[10]
pHF	*asd* ^+^, operon *caf* ^+^. operon *caf* regulated by PM.	This study
pSMA	*asd* ^+^, gene *caf1* ^−^. *caf1Mcaf1A* regulated by PM.	This study
pSA	*asd* ^+^, gene *caf1* ^−^, *caf1M* ^−^. *caf1A* regulated by PM.	This study
pSM	*asd* ^+^, gene *caf1* ^−^, *caf1A* ^−^. *caf1M* regulated by PM.	This study
pSF1	*asd* ^+^, gene *caf1* ^+^, *caf1M* ^−^, *caf1A* ^−^. gene *caf1* regulated by PM.	This study

**Figure 2:**
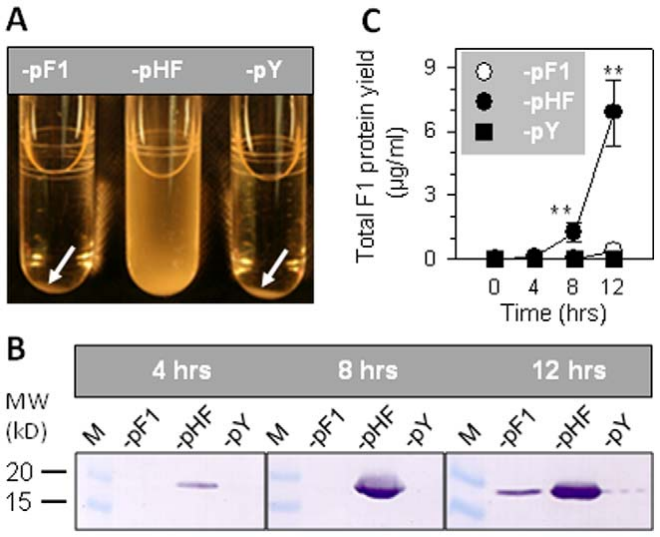
Effects of overexpression of F1 capsule on *Salmonella* phenotypes. (**A**) Overexpression of F1 capsule resultant in anti-agglutination phenotype of *Salmonella* bacilli. After centrifugation, P1-pF1 and -pY were pelleted to the tube bottom while -pHF was not. (**B**) Detection of F1 capsular expression by Western blotting. Strain P1-pF1 did not express detectable F1 capsular proteins until 12 hrs post-inoculation, but F1 capsular proteins were visible from -pHF as early as 4 hrs post-inoculation. Control P1-pY did not produce F1 capsule. (**C**) Quantification of F1 capsule. The F1 capsular protein yields of P1-pF1 and -pHF were determined and their differences were analyzed using the Tukey Kramer multiple comparisons test with ** *P*<0.01. Depicted are the mean ± SEM of three independent experiments.

These three strains were grown in LB media at 37°C with agitation at 150 rpm for determination of growth rates. During lag and stationary growth phases, no differences in growth were observed among P1-pF1, -pHF, and -pY. However, P1-pF1 and -pHF proliferated slightly more slowly than -pY, and -pHF proliferated slightly more slowly than -pF1 in logarithmic (log) phase (Figure 1D and E), suggesting that encapsulation causes negligibly delayed multiplication. Overnight cell cultures of P1-pF1 and -pY formed pellets after centrifugation, while -pHF did not (Figure 2A), implying -pHF produces enormous F1 capsular proteins that prevent cells from agglutination. Western blot analysis confirmed that the fusion promoter enhanced F1 protein expression (Figure 2B) by ∼35.2-fold and 18.8-fold in P1-pHF samples compared to -pF1 at 8 and 12 hrs post-inoculation, respectively (Figure 2C).

### Strain P1-pHF is highly attenuated in macrophages and mice

**Figure 3:**
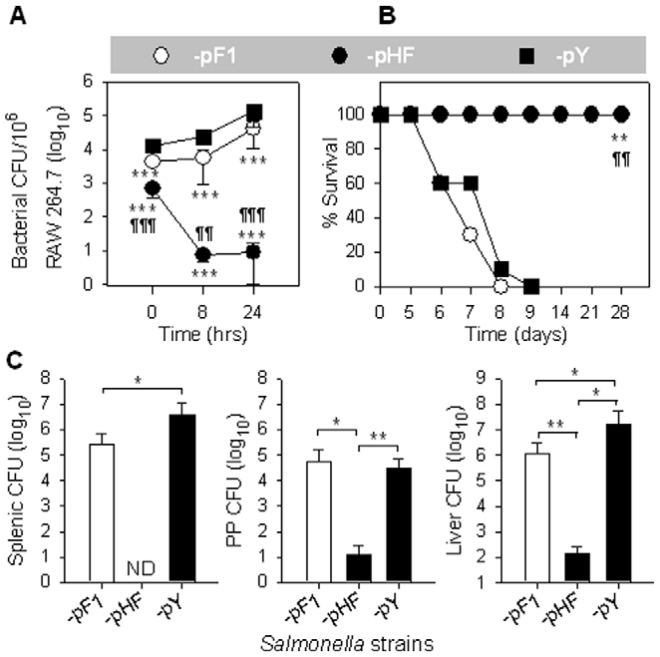
Effect of overexpression of *caf* operon on wt *Salmonella* virulence. (**A**) Caf1-mediated *Salmonella* attenuation *in vitro*. Strains P1-pF1, -pHF, and -pY were used for infecting RAW264.7 cells at a ratio of 1∶1. Bacteria recovered from macrophages were determined and statistically evaluated using the Tukey Kramer multiple comparisons test with *** *P*<0.001 for P1-pF1 or -pHF vs -pY; and ^¶¶^ *P*<0.01 and ^¶¶¶^ *P*<0.001 for -pHF vs -pF1. Depicted are the mean ± SD of three independent experiments. (**B**) Caf1-attenuated *Salmonella* was nonlethal to mice. Survival fractions of P1-pHF-administered mice (n = 9) vs -pF1 (n = 10) or -pY (n = 10) dosed mice were obtained and statistically evaluated using Mantel-Haenszel test with ** *P*<0.01 for -pHF vs -pY and ^¶¶^ *P*<0.01 for -pHF vs -pF1. Depicted are results from two independent experiments. (**C**) Caf1-attenuated *Salmonella* was unable to colonize mice. At 4 days post oral administration with 1.0×10^9^ CFUs of P1-pF1, -pHF, and -pY, mouse splenic, PP, and liver bacterial CFUs were determined and statistically evaluated using the Tukey Kramer multiple comparisons test with * *P*<0.4 and ** *P*<0.2. ND, not detected. Depicted are the mean ± SEM of two independent experiments.

To determine whether *Salmonella* encapsulation results in elevated resistance to phagocytosis, a macrophage RAW264.7 cell infection assay was performed. RAW264.7 macrophages were infected with P1-pF1, -pHF, and -pY (Figure 3A). At 0 hr, significantly fewer *Salmonella* bacilli were recovered from macrophages infected with P1-pHF or -pF1 than from those infected with -pY (Figure 3A), suggesting expression of the F1 capsule inhibits entry into macrophages, in agreement with previous findings [12]. Surprisingly, the intracellular P1-pHF could not survive within macrophages, while -pF1 proliferated in macrophages, but exhibited significant defects compared to -pY at 8 and 24 hrs post-infection (Figure 3A).

To test whether the *in vivo* virulence of the *Salmonella* was affected by F1 encapsulation, we performed a virulence assay in mice. BALB/c mice were observed for survival after they were infected with 1.0×10^9^ colony forming units (CFUs) of P1-pHF, -pF1, or -pY via oral route. All mice given P1-pHF survived (9/9), while none of the -pF1 or -pY-infected mice survived (0/10 and 0/10, respectively) (Figure 3B). These results further demonstrate that overexpression of *caf* operon attenuates *Salmonella in vivo*.

To address the mechanism by which mice survived from the encapsulated wt *Salmonella*, we performed an experiment to analyze the capability of P1-pHF to colonize mouse tissues using -pF1 and -pY as controls. The BALB/c mice were given P1-pHF, -pF1, or -pY with dose and route identical to the above survival assay. At 4 days post-administration, spleens, Peyer's patches (PP), and livers were analyzed for bacterial CFUs (Figure 3C). Strain P1-pHF was not able to colonize the spleen. Colonization of PP by P1-pHF was reduced by 2,492-fold and 5,417-fold when compared to the -pY and -pF1 infected mice, respectively, while colonization of liver by P1-pHF was reduced by 123,580-fold and 9,965-fold when compared to the -pY and -pF1 infected mice, respectively. This study clearly demonstrated that P1-pHF is highly disabled and unable to disseminate and replicate within murine tissues.

### The gene in the *caf* operon responsible for *Salmonella* attenuation is determined

**Figure 4:**
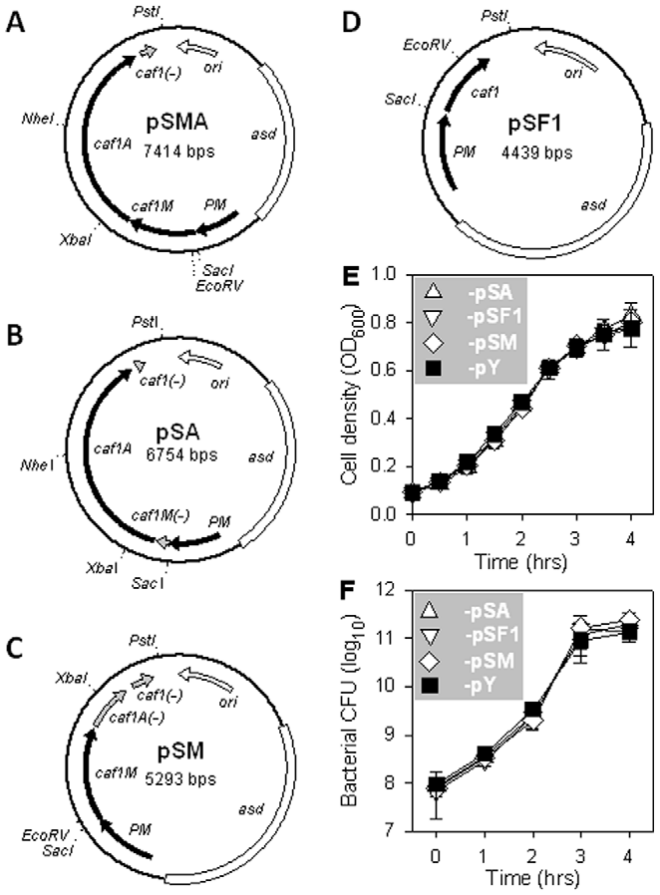
Schematic maps of plasmid pSMA, pSA, pSM, and pSF1 and growth rates of *Salmonella* harboring these plasmids. (**A**) pSMA was generated by deletion of *caf1* gene from pHF. (**B**) pSA was constructed by deletion of *caf1M* gene from pSMA. (**C**) pSM was created by deletion of *caf1A* gene from pSMA. (**D**) pSF1 was produced by cloning *caf1* gene from pF1 to pHF to replace the *caf1Mcaf1Acaf1* genes. The *caf1*(-), *caf1M*(-), and *caf1A*(-), respectively, indicate that the inner DNA sequences of gene *caf1*, *caf1M*, and *caf1A* were in-frame deleted. (**E, F**) Comparison of growth rates of P1-pSA, -pSM, -pSF1, and -pY. The bacterial growth rates were determined by measuring the OD_600_ every half hour (**E**) or determining bacterial CFU every hour (**F**), and the statistical differences of the growth rates of these strains were calculated using the Tukey Kramer multiple comparisons test. No significant differences of growth rates were discerned among these four strains.

**Figure 5:**
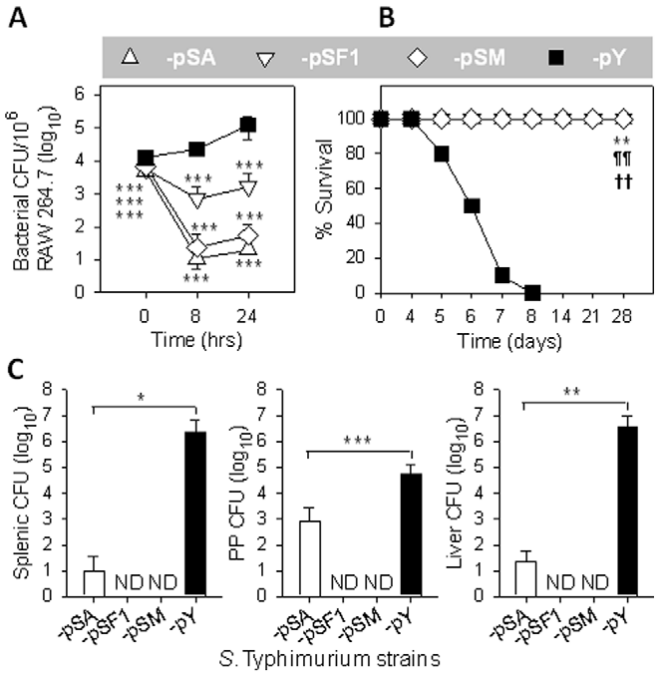
Effect of overexpression of *caf* genes on wt *Salmonella* virulence. (**A**) Overexpression of any *caf* genes was able to inactivate wt *Salmonella in vitro*. Strains P1-pSA, -pSF1, -pSM, and -pY were used for infecting RAW264.7 cells. Bacteria recovered from macrophages were determined and statistically evaluated using the Tukey Kramer multiple comparisons test with *** *P*<0.001 for P1-pSA, -pSF1, or -pSM vs -pY. Depicted are the mean ± SD of three independent experiments. (**B**) Overexpression of any *caf* genes resultant in *Salmonella* nonlethal to mice. Mice orally dosed with 1.0×10^9^ CFUs of P1-pSA, -pSF1, and -pSM all survived, while those dosed with -pY all succumbed to infection. Survival fraction obtained from P1-pSA, -pSF1, or -pSM-dosed mice was compared with -pY-dosed mice and significance was respectively determined: ** *P*<0.05, ^¶¶^ *P*<0.01, and ^††^ *P*<0.01. Each group had total of 10 mice. Depicted are the mean of two independent experiments. (**C**) Overexpression of any *caf* genes resultant in *Salmonella* unable to colonize mice *in vivo*. At 4 days post oral administration with 1.0×10^9^ CFUs of P1-pSA, -pSF1, -pSM, or -pY, mouse splenic, PP, and liver bacterial CFUs were determined and statistically evaluated using the Tukey Kramer multiple comparisons test with * *P*<0.4, ** *P*<0.2, and *** *P*<0.1. ND, not detected. Depicted are the mean ± SEM of two independent experiments.

Among the three *caf* genes harbored in pHF (Figure 1), we sought to determine the one responsible for the observed *Salmonella* attenuation. To this end, the capsular gene *caf1*, chaperone-like gene *caf1M*, and usher gene *caf1A* were deleted in-frame from pHF, and the plasmids pSA, pSF1, and pSM were created for overexpression of Caf1A, F1, and Caf1M, respectively (Figure 4). Compared to P1-pY, the -pSA, -pSF1, and -pSM strains showed no differences in growth rates (Figure 4E and 4F), but they all lost the ability to proliferate within macrophages (Figure 5A, *P*<0.001 for these 3 strains at any time points). All mice given P1-pSA (10/10), -pSF1 (10/10), or -pSM (10/10) survived, unlike all -pY-infected mice (0/10), which succumbed to infection (Figure 5B, *P*<0.01 for all these 3 strains vs control). Furthermore, strain P1-pSA only slightly colonized mouse spleen, PP, and liver, whereas -pSF1 and -pSM were undetectable in spleen, PP, and liver (Figure 5C). These results suggest overexpression of any *caf* gene can attenuate the bacterial host.

### The mechanism of *caf1A*-, *caf1*-, *caf1M*-, and *caf* operon-mediated wt *Salmonella* attenuation is analyzed

**Figure 6:**
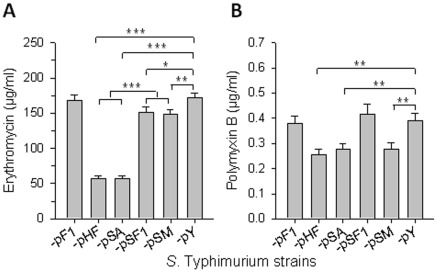
Effect of overexpression of *caf* operon or individual *caf* genes on *Salmonella* antibiotic sensitivity. Strains P1-pF1, -pHF, -pSA, -pSF1, -pSM, and -pY were analyzed for their sensitivity to (**A**) erythromycin and (**B**) polymyxin B via determination of their MICs. The MIC differences were determined and statistically evaluated using the Tukey Kramer multiple comparisons test with * *P*<0.05, ** *P*<0.01, and *** *P*<0.001. Depicted are the mean ± SEM of three independent experiments.

We hypothesized that the observed *Salmonella* attenuation due to the overexpression of genes *caf1A*, *caf1*, *caf1M*, or the *caf* operon might result from augmented membrane permeability. To test this hypothesis, strains P1-pF1, -pHF, -pSA, -pSF1, and -pSM were analyzed for sensitivity to erythromycin and polymyxin B (PMB), with -pY as a control. Erythromycin does not efficiently cross the outer membrane unless there is a breach of outer membrane integrity [18], while PMB exhibits elevated detrimental effects on the compromised bacterial outer membrane [19]. Results showed that the erythromycin minimum inhibitory concentration (MIC) of strain P1-pF1 was not different from control -pY (Figure 6A). However, the MICs of strains P1-pHF, -pSA, -pSF1, and -pSM were all significantly lower than -pY. This result correlated with the observation that P1-pF1 maintains robust virulence similarly to that of -pY (Figure 3), while -pHF, -pSA, -pSF1, and -pSM are attenuated when compared with -pY (Figure 5).

Due to the increased susceptibility to erythromycin, we speculated that P1-pHF, -pSA, -pSF1, and -pSM might also exhibit enhanced sensitivity to other antibiotics that target the cell membrane since, the antibiotic which permeabilizes the membrane may assist in further disrupting the integrity of the membrane. PMB is an antibiotic that is able to permeabilize the bacterial outer membrane and induce bactericidal activity [20]. The result showed that the PMB MICs of strains P1-pF1 and -pSF1 exhibited no differences in comparison with control -pY, while -pHF, -pSA, and -pSM displayed significantly lower MICs compared to -pY. Therefore, this experiment provides further evidence that the *Salmonella* attenuation may result from the disruption of the membrane.

### The overexpression of genes *caf1A*, *caf1*, or *caf1M* impacts *Y. pestis*

Since overexpression of *caf* operon or its individual genes can attenuate heterologous bacterium *Salmonella*, we questioned whether their overexpression also exhibits unfavorable effects on the homologous bacterium *Y. pestis*. Thus, plasmid pF1, pHF, pSA, pSF1, pSM, and pY, carrying a chloramphenicol resistance marker, were introduced to the avirulent strain *Y. pestis* KIM6+ [21], which was generated by curing the virulent plasmid pCD1 from KIM-10 [22].

**Figure 7:**
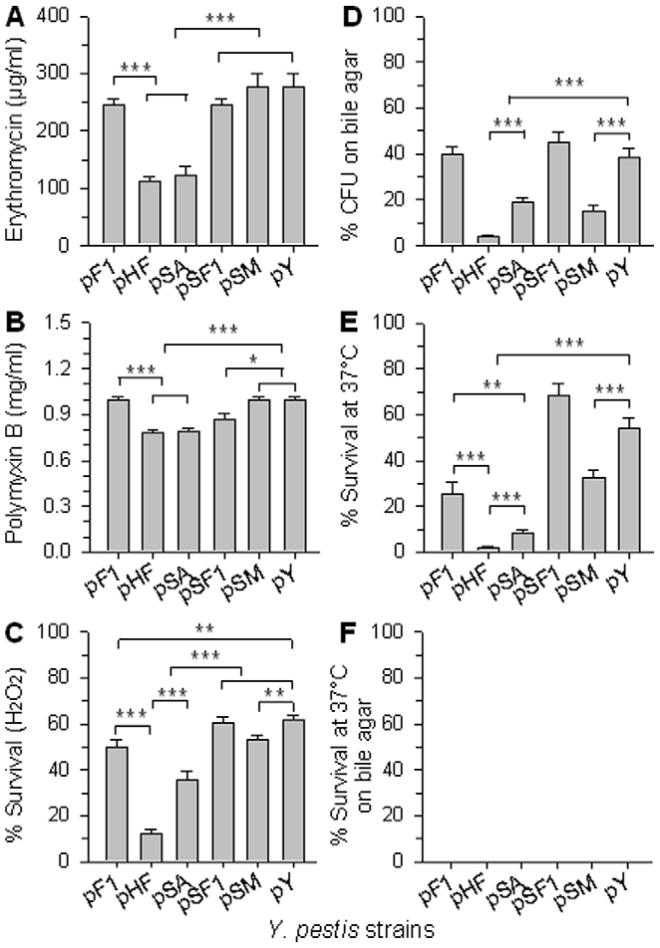
Effect of overexpression of *caf* operon or individual *caf* genes on *Yersinia* antimicrobial and temperature susceptibilities. Six strains of KIM6+/pF1, pHF, pSA, pSF1, pSM, and pY were analyzed for their sensitivities to (**A**) erythromycin, (**B**) PMB, (**C**) hydrogen peroxide, (**D**) bile salt, (**E**) temperature of 37°C, and (**F**) bile salt combined with temperature of 37°C. (**A**, **B**) The erythromycin and PMB MICs of the 6 strains were respectively determined. (**C**) The 6 strains were incubated with 2.5 mM H_2_O_2_ for 1 hr and the survival percentages were determined in comparison with the non-treated controls, respectively. (**D**) The 6 strains were dropped onto BHI agar containing 1% bile and were incubated at 27°C for 48 hrs for CFU enumeration in comparison with those grown on BHI agar without bile, respectively. (**E**) The 6 strains were dropped onto BHI agar and were incubated at 37°C for CFU enumeration in comparison with those grown on BHI agar at 27°C, respectively. (**F**) The 6 strains were dropped onto BHI agar containing 1% bile and were incubated at 37°C for CFU enumeration in comparison with those grown on BHI agar without bile at 27°C, respectively. All experiments (**A** to **F**) were statistically analyzed for significant differences among these 6 strains using the Tukey Kramer multiple comparisons test with * *P*<0.05, ** *P*<0.01, and *** *P*<0.001. Depicted (**A** to **F**) are the mean ± SEM of three independent experiments.

Strains KIM6+/pF1, pHF, pSA, pSF1, pSM, and pY were subjected to antimicrobial and temperature sensitivity assays. The results showed that KIM6+/pSA is susceptible to erythromycin (Figure 7A), PMB (Figure 7B), hydrogen peroxide (Figure 7C), bile (Figure 7D), and is unable to vigorously grow at 37°C (Figure 7E) relative to control pY. Strain KIM6+/pSF1 is sensitive to PMB (Figure 7B), and pSM is labile to peroxide hydrogen (Figure 7C), bile (Figure 7D), and high temperature (Figure 7E) when compared to pY. These observations suggest that KIM6+/pSA is the most delicate among the three individual genes recombinant strains, and KIM6+/pHF is even more fragile than strain pSA. In fact, KIM6+/pHF exhibited the most sensitive phenotypes among all the tested strains and all the tested stress conditions (Figure 7A–E). Strain KIM6+/pF1 was slightly sensitive to both hydrogen peroxide (Figure 7C) and high temperature (Figure 7E). Interestingly, all 6 strains, KIM6+/pF1, pHF, pSA, pSF1, pSM, and pY, were able to survive at 27°C on Brain Heart Infusion (BHI) agar with bile (Figure 7D) or at 37°C without bile (Figure 7E), but none survived at 37°C on BHI agar with bile (Figure 7F). This suggests that the double pressures from bile and the high temperature are lethal to all the recombinant strains, as well as the control. This clearly indicates overexpression of individual genes of *caf1A*, *caf1*, *caf1M*, or the whole *caf* operon produces strikingly deleterious effects to the homologous bacterium *Y. pestis*.

**Figure 8:**
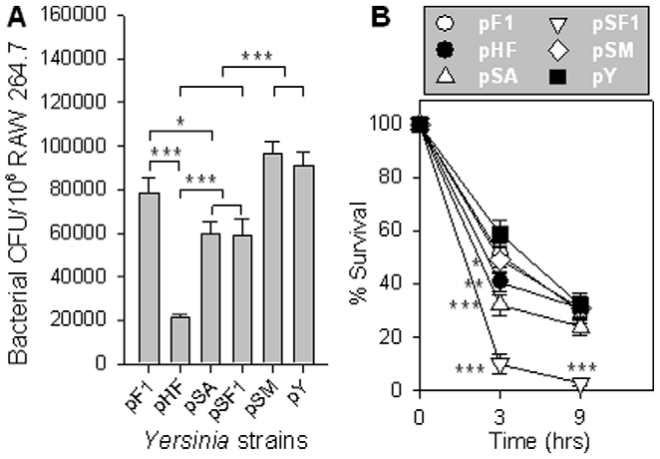
Macrophage infection and survival assays of the recombinant *Y. pestis* KIM6+ strains. (**A**). infectivity assay. Strains KIM6+/pF1, pHF, pSA, pSF1, pSM, and pY were used for infecting macrophage RAW264.7 cells. At 1 hr post-infection, the intracellular bacterial CFUs were compared among these 6 strains. The Tukey Kramer multiple comparisons test was used for significant difference calculation with * *P*<0.05, ** *P*<0.01, and *** *P*<0.001. Depicted are the mean ± SEM of three independent experiments. (**B**) Survival assay. Bacteria survived within the macrophages at 3 and 9 hrs post-infection were compared with the 0 hr CFU counts, and the percentages were calculated. Differences in survival rates among the 6 strains were calculated using the Tukey Kramer multiple comparisons test. Values depict the mean ± SEM of three independent experiments with * *P*<0.05, ** *P*<0.01, and *** *P*<0.001 for the *caf* operon or *caf* gene expression strains vs control KIM6+/pY.

Subsequently, the infection abilities and survivability of these strains were determined using macrophage RAW264.7 cells. KIM6+/pSA and pSF1 lost partial capability of infecting macrophages, but pSM did not when compared to control pY (Figure 8A). As expected, KIM6+/pHF displayed the lowest infectivity among all the tested strains, while pF1 showed no differences from pY (Figure 8A). This suggests that overexpression of either *caf1A* or *caf1*, particularly the entire *caf* operon, dramatically reduces the bacterial infectivity. KIM6+/pSF1 was cleared from macrophage very rapidly after infection. By 3 and 9 hrs post-infection, only 10.0% and 3.1% of the initial population of KIM6+/pSF1 remained alive, respectively (Figure 8B). Strain KIM6+/pSA, pHF, and pF1 also showed impaired survivability when compared to pY, while pSM exhibited no differences from pY. These results explicitly suggest that the *caf* operon and its individual gene *caf1A* and *caf1* possess severe deleterious effects to *Y. pestis*.

## Discussion

Since *Y. pestis* is classified as a category A select agent by the CDC [23], manipulation of wt *Y. pestis* requires a Biosafety Level 3 (BSL-3) facility, which limits access to and work with this pathogen. Thus, in our study, we established a platform for analyzing *Y. pestis* virulence factors by means of the wt BSL-2 pathogen, *S.* Typhimurium. As the *caf* operon has previously been demonstrated to be functional in *Salmonella* [10], use of wt *Salmonella* strains to analyze *Yersinia* virulence factors is feasible.

To investigate whether *caf* possesses deleterious aspects during infection, we amplified the expression of *caf* operon in order to maximally expose these effects. Hence, the *caf* native promoter P*caf1M* was replaced with a strong fusion promoter, which increased F1 protein yield by 18.8- to 35.2-fold (Figure 2C). Results showed that overexpression of the *caf* operon heavily attenuates *Salmonella*, which was evidenced by P1-pHF being unable to proliferate in macrophages (Figure 3A) or colonize mouse spleens (Figure 3C), a similar attenuation impact only seen when highly virulent genes, such as *phoP* and *rfaC*, are deleted from the *Salmonella* genome [24]. This clearly demonstrates that potently deleterious effects are indeed encoded by the *caf* operon, as amplification of the *caf* operon renders the bacterium markedly attenuated *in vitro* and *in vivo*. To circumvent the deleterious effect of *caf*, *Y. pestis* cleverly uses a weak and inducible promoter, P*caf1M*, to precisely govern *caf* operon expression. Through P*caf1M*, the vulnerability associated with the *caf* operon is adequately camouflaged allowing the P1-pF1 to still possess similar virulence to -pY *in vivo*.

As there are three structural genes encoded in the *caf* operon, we queried which one was responsible for the *caf* operon-mediated *Salmonella* attenuation. Our data showed that overexpression of any genes of the *caf* operon, including chaperone-like protein encoding gene *caf1M*, outer membrane protein encoding gene *caf1A*, and the capsule encoding gene *caf1*, was able to undermine the virulence of *Salmonella* (Figure 5). We then investigated the mechanisms of why overexpression of these individual genes, as well as the *caf* operon, could attenuate *Salmonella*. Seemingly, the impairments induced by overexpression of *caf* or its components *in vitro* and *in vivo* were associated with the elevated cell membrane permeability (Figure 6). When compared with P1-pY, the erythromycin MICs were significantly decreased for all strains other than P1-pF1, suggesting that -pF1 carefully regulates its vulnerability. The erythromycin MIC for strain P1-pSA expressing *caf1A* alone was identical to -pHF, suggesting these two strains share similar membrane permeability. The reason P1-pSA and -pHF possess similar membrane permeability is possibly due to the usher Caf1A alone being able to self-assemble into functional channels in the outer membrane without the assistance of the chaperone Caf1M. The over-installed Caf1A channels in the outer membrane facilitated the free entrance of the large molecule erythromycin and resulted in a decreased MIC. The Caf1A impairment to the membrane may dominate the overall detrimental effects associated with the *caf* operon shadowing the impairment of the Caf1M and F1 proteins, to the cell membrane. Thus, P1-pSA and -pHF display indifferent erythromycin sensitivities. Others have previously recognized that elevated curli expression increases the erythromycin sensitivity [18]. Thus, by combining previous observations with our results, we may conclude that overexpression of the cell surface appendages will proportionally enhance the channel expression level, and the elevated channels in the cell outer membrane facilitate entry of erythromycin into the cell, resulting in heightened bacterial susceptibility. Introduction of membrane permeabilizer antibiotic PMB into the channeled cell membrane [25] further increased cell permeability and resulted in decreased MICs, as evidenced by the decreased PMB MIC of strain P1-pSA and -pHF. The *Salmonella* attenuation caused by overexpression of genes *caf1M* and *caf1* may be because these proteins are arrested within the periplasm. This may result in (1) other periplasmic proteins displaying abnormal function and (2) physical injuries to the membranes when they accumulate. Both of these possibilities can be applied to Caf1M since Caf1M is naturally located in periplasm due to its signal peptide guided transportation [26]. Thus, overexpression of *caf1M* in P1-pSM led to a decreased erythromycin and PMB MICs. The above two possibilities may also apply to F1 protein. Normally, the newly synthesized F1 capsular protein monomers are transported from cytoplasm to periplasm directed by its signal peptide and further traverse outer membranes to form polymers with the assistance of Caf1A and Caf1M [27]. In the absence of Caf1A and Caf1M, F1 proteins were not able to cross the outer membrane. Hence, F1 proteins trapped within the periplasm would display detrimental effects to the membrane, as in the case of P1-pSF1. Notably, P1-pSF1 showed no elevated sensitivity to PMB (Figure 6). One possible explanation for this observation, which will require further study, is that accumulated F1 proteins in the periplasm served as a protective layer against PMB disruption of the inner membrane. Nevertheless, since F1 proteins did not accumulate in the bacterial periplasm in the presence of Caf1A and Caf1M, F1-mediated *Salmonella* impairment might occur only sporadically.

Since all three proteins overexpressed from the *caf* operon could impair *Salmonella* bacilli, we then questioned if proteins from any source could cause detrimental effects when accumulated within the periplasm. We observed no overt detrimental impact on *Salmonella* when chaperone gene *cfaA* or usher gene *cfaC* from the enterotoxigenic *Escherichia coli* (ETEC) *cfa*/I operon was overexpressed in the periplasm [17]. Thus, impairment of *Salmonella* caused by overexpression of *caf1M* or *caf1A* seemed likely to be inherent in the *caf* operon. This deduction is virtually endorsed by the impacts of these individual genes on the *Y. pestis* strain KIM6+ (Figure 7). Overexpression of *caf1A* alone resulted in considerably high susceptibility of KIM6+ to both erythromycin and PMB. In addition to erythromycin and PMB, KIM6+/pSA also exhibited increased sensitivity to hydrogen peroxide, bile, and a high temperature of 37°C. This directly led to the lowered infectivity and survival of the KIM6+/pSA (Figure 8). Overexpression of *caf1M* alone in KIM6+ did not alter bacterial sensitivity to erythromycin and PMB, but resulted in the elevated sensitivity to bile, hydrogen peroxide, and high temperature. However, the macrophage infectivity and survival assays of KIM6+/pSM did not reveal any defects relative to control pY (Figure 8). Of interest is the strain KIM6+/pSF1 only showed slight susceptibility to PMB, but showed no sensitivity to erythromycin, hydrogen peroxide, bile, and high temperature. Nevertheless, KIM6+/pSF1 displayed low infectivity similar to that of pSA, and its survival rate within macrophages was the lowest among all the recombinant *Yersinia* strains tested. This suggested the F1 antigen possessed the most potent detrimental effects *in vitro* among the three *caf* genes. As discussed above, the F1-mediated deleterious effects may not be fully implemented to *Yersinia* in the presence of the Caf1M and Caf1A, since it may be secreted out of the cells with the assistance of these two proteins [12], [28]. By using KIM6+ to analyze the *caf* genes' impact to the bacteria, we saw that these individual *caf* genes displayed a synergistically deleterious effect when co-expressed. This was demonstrated by the observation that KIM6+/pHF showed the most sensitive phenotypes to the hydrogen peroxide, bile, and high temperature among all the tested recombinant KIM6+ strains (Figure 7). Furthermore, KIM6+/pHF exhibited the lowest infecting capability compared with the other KIM6+ strains (Figure 8). This reduced infectivity may be associated with the overexpressed F1 capsular anti-phagocytic function [12], or may be associated with the following: (1) KIM6+/pHF may not be able to swim as vigorously as the other KIM6+ strains due to the viscous capsule on the cell surface. Previous study on *Pseudomonas aeruginosa* showed swimming motility is critical for the bacteria to establish a successful macrophage infection [29]. Thus, KIM6+/pHF may not effectively contact the macrophage to infect. (2) KIM6+/pHF may be neutralized by the macrophages after infection, particularly at the very early time points post-infection, i.e., during the infection incubation period. These two speculations may require a motile behavior assay and a dynamically microscopic observation for further investigation. In any case, this study implies that the vulnerability of *caf* operon found through *Salmonella* vector is applicable to the *Yersinia*.

In summary, results achieved from this study do indicate that *caf* associates with serious vulnerabilities. In addition to the previously found immunogenic property of F1 capsular proteins which may potentially render *Yersinia* to an unfavorable condition during infection, this study suggests that the Caf1 apparatus may also be an Achilles' heel when overexpressed. *Yersinia* benefits from *caf* operon expression via anti-phagocytic role of F1 capsule; however, it runs the risk of exposing the deleterious effects rooted in the Caf1 apparatus. Thus, the Caf1 apparatus may serve as a double-edged sword for *Yersinia*. *Yersinia* cautiously manages a balance between the advantages and disadvantages conferred by *caf* through regulating its expression with an elaborate promoter P*caf1M*, which determines when and where it should switch on and most importantly to what level it should transcribe, in order to take the advantage of F1 capsule while preventing the deleterious effects from being exposed.

## Materials and Methods

### Bacterial strains, media, and molecular manipulations

The bacterial strains, plasmids, and their relevant characteristics are provided in Table 1. The primers used for polymerase chain reaction (PCR) are listed in Table 2. Diaminopimelic acid (DAP) (50 µg/ml) was used for *E. coli* H681 or *S.* Typhimurium P1 culture unless a plasmid containing *asd* was introduced. The *asd* ^+^ plasmids pHF, pSA, pSF1, and pSM were constructed by means of H681 and transformed to P1 to generate *Salmonella* recombinant strains. Lysogeny broth (LB; 10 g of tryptone, 10 g of NaCl, and 5 g of yeast extract per liter) was used for recombinant *E. coli* and *Salmonella* growth at 37°C without DAP supplementation. Bacteria were cultured in LB and stored at −80°C in LB plus 20% glycerol. For growth rate comparison among the recombinant *Salmonella* strains, cells were inoculated from −80°C stock to LB agar for overnight incubation at 37°C. *Salmonella* bacilli were collected from plates, and the cell optical density at 600 nm (OD_600_) was adjusted to ∼0.1. Bacterial inoculants were subjected to culture for 4 hrs at 37°C in BioScreen C (Lab Systems) with agitation at 150 rpm, and OD_600_ was measured every half hour. Data were downloaded and utilized for statistical comparison among these strains. Meanwhile, the bacterial CFUs were determined at one-hour intervals via colony enumeration after they were made serial dilutions on LB agar plates and incubation overnight at 37°C.

**Table 2:** Primer sequences and restriction enzyme sites integrated.

Primer names	Enzyme sites	Primer sequences^b^^,^ c
caf-F	SacI	**GAGCTC**CGTAAGGAGGTTAAGC
caf-R	PstI	**CTGCAG**TGAACCTATTATATTGCTTCGCGC
F1-dn-F	EcoRV	**GATATC**GTAACCGTATCTAACCAATAATCC
F1-dn-R	PstI	**CTGCAG**TGAACCTATTATATTGCTTCGCGC
F1-up-F	NheI	ATCGTTAAACATT**GCTAGC**GAGGAATACGCC
F1-up-R	SmaI	**CCCGGG**AGTGGTGCTTGCAGTTAAATCTG
M-dn-F	SmaI	**CCCGGG**TTGGATCGTTTGTATTCC
M-dn-R	XbaI^a^	TTGTAAGGATGATAGGCATGGC
A-dn-F	XbaI	**TCTAGA**CGGTGTCTATTTGACTGGACTAC
F1-F	SacI	**GAGCTC**ATTATTCGATAGAGGTAATATATG

Note:

a The restriction enzyme site XbaI locates downstream of this primer.

b The sequences of the restriction enzyme sites integrated in the primers are bolded.

c Primer sequences are based on template of *Y. pestis* plasmid pFra (accession No. X61996).

To determine whether overexpression of *caf* operon or *caf* individual genes of *caf1A*, *caf1*, and *caf1M* impact the homologous bacterium *Y. pestis*, plasmids pF1, pHF, pSA, pSF1, pSM, and pY were transferred to KIM6+ after they were modified with a chloramphenicol resistance marker. Strain *Y. pestis* KIM6+ was a gift kindly provided by Robert D. Perry (University of Kentucky, Lexington, Kentucky, USA). The conditions used for growing KIM6+ derived strains were 27°C without shaking in BHI liquid medium unless otherwise indicated.

Restriction endonucleases, T4 DNA ligase, and Taq DNA polymerase were purchased from New England BioLabs (Beverly, MA) unless otherwise noted. Chemicals were purchased from Sigma-Aldrich (St. Louis, MO). Genetic manipulations were conducted by using the method as described elsewhere [10]. Plasmid DNA was extracted by using a Qiagen Miniprep Kit (Valencia, CA). DNA fragments were purified and extracted from agarose gel slices, using Qiagen Gel Extraction Kit. Competent *E. coli* and *S.* Typhimurium cells were made in 10% glycerol and transformed by electroporation.

### Construction of plasmids

#### Plasmids pHF and pY

The genes of *caf1Mcaf1Acaf1* from plasmid pF1 [10] were amplified with the primers of caf1-F and caf1-R (Table 2), and the DNA fragment was Topo cloned (Invitrogen, Carlsbad, CA). After sequencing for identification with the original *caf* DNA sequence, the correct clone was used for subcloning. The *caf1Mcaf1Acaf1* DNA fragment with a length of 4153 bps was placed downstream of promoter PM of plasmid pHC [16] to generate pHF (Figure 1B). Plasmid pY was derived from pHC by using ScaI to remove the *cfa/I* operon, and the vector fragment was then self-ligated (Figure 1C).

#### Interim plasmid pSMA

Prior to pSA and pSM construction, an interim plasmid pSMA was constructed (Figure 4A). Plasmid pSMA was derived from pHF by deleting its *caf1* gene inner DNA sequence. The *caf1* gene downstream sequence was amplified with a pair of primers of F1-dn-F+F1-dn-R, and its upstream sequence was amplified with another pair of primers of F1-up-F+F1-up-R (Table 2). The two DNA fragments were subjected to Topo cloning and sequencing to confirm no mutation was introduced, and then they were fused at the EcoRV and SmaI sites since both are blunt ends. A total of 405 bps was deleted from *caf1* gene, and this fusion DNA fragment was placed between NheI and PstI sites in pHF. This plasmid was termed pSMA. Based on pSMA, the plasmids pSA and pSM were constructed as follows:

#### Plasmids pSA and pSM

The DNA sequence downstream *caf1M* was amplified with a pair of primers of M-dn-F+M-dn-R (Table 2). After sequencing identification, it was digested with SmaI and XbaI, and this 287 bps DNA fragment was inserted between the EcoRV and XbaI sites in pSMA. Thus, the 660 bps inner DNA sequence of *caf1M* was deleted in-frame. The new plasmid was termed pSA (Figure 4B). The DNA sequence downstream *caf1A* was amplified with a pair of primers of A-dn-F+caf1-R (Table 2). After sequencing confirmation and digested with XbaI and PstI, this 993 bps DNA fragment was installed between the XbaI and PstI sites in pSMA. The 2121 bps inner DNA sequence of *caf1A* was deleted in-frame. This new plasmid was termed pSM (Figure 4C).

#### Plasmid pSF1

The *caf1* gene from pF1 was amplified by a pair of primers of F1-F+caf1-R (Table 2). Fidelity of the 773 bps *caf1* gene was verified by sequencing, and it was then inserted into pHF between SacI and PstI sites. Thus, the 4153 bps *caf* operon was replaced by the *caf1* gene. The new plasmid was termed pSF1 (Figure 4D).

### Western blot analysis of Caf1 expression

To verify F1 protein expression and yield, P1-pF1, -pHF, and -pY were inoculated from −80°C freezer to LB agar and allowed to grow overnight at 37°C. Bacteria were then inoculated into liquid LB media with the cell OD_600_ adjusted to ∼0.1. They were incubated at 37°C with shaking at 150 rpm. At 4, 8 and 12 hrs post-inoculation, cultures were centrifuged at 13,000 g for 15 min at 4°C to collect whole cells. Secreted F1 capsular proteins contained in supernatants were precipitated by ammonium sulfate added to 30% saturation as previously described [30]. Supernatant protein pellets were mixed with their corresponding whole cell pellets of P1-pF1, -pHF, and -pY. Samples were subjected to Western blot analysis as previously described [10], and each well was loaded with an equivalent number of CFUs of P1-pF1, -pHF, and -pY cells. The F1 protein yields were quantified by densitometric analysis as previously described [31].

### Evaluation of survivability of recombinant *Salmonella* and *Yersinia* strains in RAW264.7 macrophages

RAW264.7 macrophage cell line (American Type Culture Collection) was used for evaluating the capability of infection and replication for strains P1-pF1, -pHF, -pSA, -pSF1, -pSM, and -pY. Infections were conducted as previously described [32]. 1.25×10^6^ RAW264.7 cells/well without antibiotics were allowed to adhere to plastic in 24-well microtiter dishes (BD-Labware, Franklin Lakes, NJ) at 37°C with 5% CO_2_. Wells were washed, and the nonadherent cells were collected and counted to determine cell numbers that remained plastic-adherent. After overnight culture, cells were infected with bacteria to macrophage ratio of 1∶1 for 1 hr at 37°C. Wells were washed twice and then incubated with 50 µg/ml of gentamicin for 30 minutes at 37°C. After washing twice, as described, fresh complete medium containing 2.5 µg/ml of gentamicin was added, and cells were incubated for either an additional 8 or 24 hrs. Next, the macrophages were water lysed, and the bacteria were placed on LB agar plates after a series of dilutions. After incubation overnight at 37°C, the bacterial CFUs were determined.

To determine whether the recombinant *Yersinia* strains of KIM6+/pF1, pHF, pSA, pSF1, and pSM were capable of surviving as robustly as pY *in vitro*, a macrophage infection assay was conducted as previously described [33]. Briefly, log phase cultures of KIM6+/pF1, pHF, pSA, pSF1, pSM, and pY were adjusted so the bacteria to macrophage infection ratio was 1∶1. Wells were washed twice, and then incubated with 8 µg/ml of gentamicin for 1 hr at 37°C. After washing twice, as described, fresh complete medium containing 2 µg/ml of gentamicin was added, and cells were incubated for an additional 3 or 9 hrs. The infected macrophages that were not supplied with the 2 µg/ml gentamicin were water lysed for bacterial CFU enumeration, and the bacterial CFUs (defined time point, 0 hr) were compared among the 6 strains to determine their infection capabilities. The bacterial CFUs obtained at 0, 3, and 9 hrs post-infection were then calculated for bacterial macrophage survival assay with the initial bacterial counts (t = 0) as 100%. After water lysis, the released bacteria were placed on BHI agar plates and incubated for 48 hrs at 27°C, and the bacterial CFUs were enumerated.

### Mouse studies

Pathogen-free female BALB/c mice (National Cancer Institute, Frederick Cancer Research Facility) of 7–9 wks of age were used throughout this study. All mice were maintained at Montana State University Animal Resource Center under pathogen-free conditions in individually ventilated cages under HEPA-filtered barrier conditions and were fed sterile food and water *ad libitum*. All animal care and procedures were in accordance with institutional policies for animal health and well-being.

To assess the virulence of the newly constructed P1-derived strains, groups of BALB/c mice (5–7 individuals/group) were orally gavaged with 1.0×10^9^ CFUs of P1-pF1, -pHF, -pSA, -pSF1, -pSM, and -pY in 200 µl sterile phosphate buffered saline (sPBS). Mice were monitored for mortality for 4 wks post-administration, and experiment was repeated twice. For tissue colonization assay, mice were sacrificed at 4 days post-administration. Spleen, PP, and liver were excised under aseptic conditions for determination of bacterial burden as previously described [17], [34].

### Antimicrobial susceptibility tests

The recombinant *Salmonella* strains of P1-pF1, -pHF, -pSA, -pSF1, -pSM with control -pY were determined for MIC under aerated conditions. They were subjected to various concentrations of erythromycin and PMB in polypropylene microtiter plates (Costar Corp., Cambridge, MA) at 37°C in LB medium. MIC was calculated according to the method as previously described [35].

The KIM6+/pF1, pHF, pSA, pSF1, pSM, and control pY were determined for erythromycin and PMB MICs using the procedure as previously described with slight modifications [36]. *Y. pestis* strains KIM6+/pF1, pHF, pSA, pSF1, pSM, and pY were grown in BHI broth at 27°C for 18 hrs without shaking, since we found that shaking would result in serious cell agglutination for all these 6 strains (data not shown). The cell cultures were then used for evaluating in MIC assays. In the preliminary experiment, we found that by using the cell density of 10^5^ CFU/ml [36] we were unable to detect any bacterial growth after 48 hrs incubation at 27°C in 96-well polypropylene microtiter plates, even though no antibiotics were added in the medium. Thus, we raised the cell density by 100-fold (final cell concentration, 10^7^ CFU/ml) for the MIC determination. At this cell density, the MICs of erythromycin and PMB for these recombinant strains were determined at 24 hrs post-incubation at 27°C. At the end of the assays, the OD_600_ of the bacterial suspensions were measured by using a multichannel plate reader BioScreen C. The MICs were determined as the lowest concentrations of erythromycin and PMB, which did not result in measurable growth at the end of the experimental period [37]. Each sample was run in triplicate, and experiments were repeated three times.

The recombinant KIM6+ strains were further subjected to analysis of susceptibility to two additional antimicrobial reagents, hydrogen peroxide (H_2_O_2_) and ox bile salt (Sigma-Aldrich (St. Louis, MO). Human cells produce hydrogen peroxide as a first line of defense against bacteria pathogens [38], and the secreted bile serves as the humoral barrier, interfering with normal function of the bacterial membrane and damaging the bacterial DNA [39]. The sensitivity to H_2_O_2_ was conducted according to the previously described method [40]. H_2_O_2_ (final concentration, 2.5 mM) was added to bacteria (final cell density, 10^5^ CFU/ml) resuspended in sPBS. The mixture was allowed to incubate at 27°C for 1 hr, and the cells were briefly spun down to remove the H_2_O_2_. After a series of dilutions, the cells were placed on BHI agar for incubation at 27°C for 48 hrs, and the CFUs were enumerated. A parallel set of cultures not exposed to H_2_O_2_ served as a control. The bile sensitivity assay was performed by adding bile salt to BHI agar (final concentration, 1%) [41], and the log phase bacteria were placed onto the plates. Cells were incubated at 27°C for 48 hrs, and the CFUs were enumerated. The bacteria grown up on BHI agar without bile were used as a control. Three independent experiments were done for both hydrogen peroxide and bile assays.

### Statistical analysis

The Tukey Kramer multiple comparisons test was used for assessing differences among experimental parameters. The Kaplan-Meier method (GraphPad Prism, GraphPad Software, Inc., La Jolla, CA) was applied to obtain the mouse survival fractions following infection with recombinant *Salmonella* strains. Using the Mantel-Haenszel log rank test, the *P*-values for statistical differences between inactivated *Salmonella* strains and control *Salmonella* strain-dosed mice were discerned at the 95% confidence interval.
